# Ranolazine in patients with chronic coronary syndromes: real-world data provide new evidence on the antiarrhythmic properties of the drug

**DOI:** 10.1093/ehjcvp/pvaf074

**Published:** 2025-09-30

**Authors:** Stefano Fumagalli, Melania Dovizio, Stefania Mazzoni, Luca Degli Esposti, Emanuele Santamaria, Giulia Spanalatte, Carlo Fumagalli, Camilla Cagnoni, Arianna Tariello, Elisabetta Cerbai, Niccolò Marchionni

**Affiliations:** Department of Experimental and Clinical Medicine, Unit of Internal and Specialist Medicine, University of Florence and AOU Careggi, Viale G. Pieraccini 6, Florence 50139, Italy; Department of Innovative Technologies in Medicine & Dentistry, ‘G. D’ Annunzio’ University of Chieti-Pescara, Chieti 66100, Italy; CliCon S.r.l. Società Benefit, Health, Economics & Outcomes Research, Bologna 40137, Italy; CliCon S.r.l. Società Benefit, Health, Economics & Outcomes Research, Bologna 40137, Italy; CliCon S.r.l. Società Benefit, Health, Economics & Outcomes Research, Bologna 40137, Italy; Department of Experimental and Clinical Medicine, Geriatric Intensive Care Unit, University of Florence and AOU Careggi, Florence 50134, Italy; Department of Experimental and Clinical Medicine, Geriatric Intensive Care Unit, University of Florence and AOU Careggi, Florence 50134, Italy; University of Campania ‘Luigi Vanvitelli’, Naples 80138, Italy; Department of Experimental and Clinical Medicine, Geriatric Intensive Care Unit, University of Florence and AOU Careggi, Florence 50134, Italy; Department of Experimental and Clinical Medicine, Geriatric Intensive Care Unit, University of Florence and AOU Careggi, Florence 50134, Italy; Department of Neuroscience, Innovative Treatment, Drug Research and Child Health—‘NEUROFARBA’, Section of Pharmacology and Toxicology, University of Florence, Florence 50139, Italy; Department of Experimental and Clinical Medicine, University of Florence, Florence 50134, Italy

**Keywords:** Anti-arrhythmic therapy, Atrial fibrillation, Chronic coronary syndrome, Drug therapy, Prognosis, Ranolazine

## Abstract

**Aims:**

Ranolazine (Ran) is an anti-anginal drug inhibiting late sodium current, an action possibly hindering arrhythmias onset. Indeed, some evidence supports the anti-arrhythmic effects of Ran. The aim of this study, which evaluated Italian patients with chronic coronary syndrome (CCS), was to investigate whether Ran, as an add-on therapy, was associated with a lower incidence of atrial fibrillation (AF) compared with no-Ran prescription (No-Ran).

**Methods and results:**

The original population (*N* = 6.1 million) derived from the databases of the Italian National Health System; information concerned hospitalizations with the related diagnoses, drug therapy, follow-up clinical events and visits. Patients hospitalized between 2011 and 2020 for any cause and discharged with an ICD-9-CM CCS code were studied if AF had not been diagnosed before. The follow-up duration was 4.4 and 5.0 years for the Ran and the No-Ran cohorts, respectively. Study subjects were 171 015 (mean age: 72 years; men: 66%; Ran: *N* = 22 207; No-Ran: *N* = 148 808). After propensity score matching, Ran (*N* = 6384) and No-Ran (*N* = 25 536) cohorts were similar for age, sex, comorbidities and drug therapy. AF incidence during follow-up was 5.3% and 9.6% in the Ran and in the No-Ran cohorts, respectively, with a 41% drug-related lower risk of arrhythmia development in the Cox model (HR = 0.59, 95% CI: 0.53–0.67, *P* < 0.001). Also, Ran correlated with reduced incidence of brady-arrhythmias (*P* = 0.001) and ventricular tachy-arrhythmias (*P* = 0.049), and with lower mortality (*P* < 0.001).

**Conclusion:**

Our study, performed in a subset of the Italian CCS population, showed that Ran therapy was safe and associated with a long-term reduced AF incidence.

## Graphical Abstract

**Graphical Abstract:**
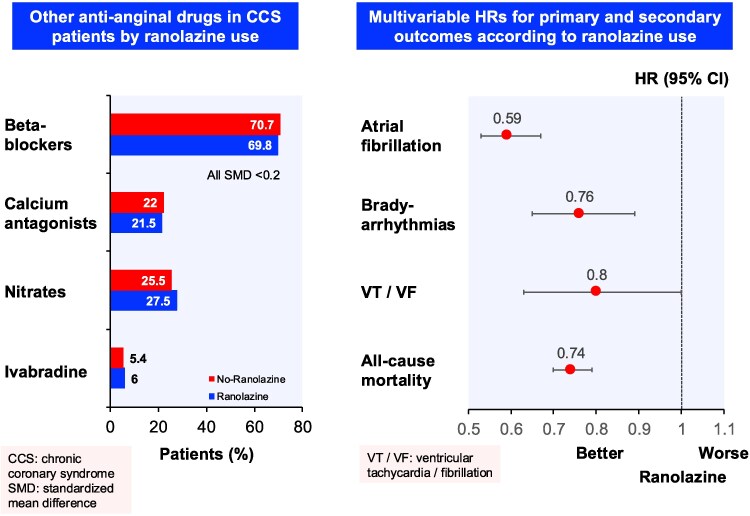
Use of ‘other’ anti-anginal drugs in the study population after propensity score matching, by ranolazine therapy. No differences were observed between groups and the drug resulted as a true add-on therapy, in accordance with current Guidelines on chronic coronary syndrome management (left). Hazard Ratios (HRs) of multivariable models exploring the association of ranolazine with primary and secondary clinical endpoints. In all cases, drug therapy was associated with a favourable outcome (right).

## Introduction

Ranolazine is a piperazine derivative recommended as an add-on therapy in patients with chronic coronary syndrome (CCS) and uncontrolled symptoms or not tolerant to standard frontline anti-anginal drugs.^1^ Ranolazine can reduce myocardial ischaemia by inhibiting the late inward Na^+^ current in cardiac myocytes during repolarization, thus hindering intracellular Na^+^ overload and the subsequent increase in cytosolic Ca^2+^ by modulating the reverse-mode Na^+^-Ca^2+^ exchange.^2^ Importantly, the late inward Na^+^ current is increased during myocardial ischaemia and the development of heart failure (HF).^2,3^ The benefits of ranolazine in reducing Ca^2+^ overload and left ventricular dysfunction during ischaemia/reperfusion was first proven in rat experimental models more than 15 years ago.^4^ Hence, ranolazine initially introduced as an anti-anginal agent,^5,6^ was later shown to reduce myocardial proarrhythmic substrates and triggers,^6,7^ possibly exerting a dual protection against atrial fibrillation (AF) and ventricular arrhythmias (VA).^8^ Since the standard anti-arrhythmic agents for the treatment of AF have some limitations due to suboptimal efficacy and potential side effects, ranolazine has been then investigated as a stand-alone therapy to prevent AF recurrence,^9^ or as an add-on agent to improve the efficacy of amiodarone^10^ or dronedarone.^11,12^

In Italy, ranolazine is indicated and reimbursed by the National Health System (NHS) as a second-line therapy for the treatment of angina pectoris inadequately controlled by first-line drugs (i.e. beta-blockers and/or calcium channel blockers).

The present, real-world study used large administrative databases to investigate, in patients with CCS, the association of ranolazine with incident AF or all-cause death as primary outcomes. The risk of bradyarrhythmias or ventricular tachyarrhythmias was also explored as secondary outcomes.

## Methods

### Data source

We retrospectively analyzed administrative databases including ∼6.1 million (i.e. more than 10% of Italian population) of unselected residents assisted by Italian local health units (LHU) participating in a project of cardiovascular pharmacoepidemiology and outcome research.^13^ Such databases track all healthcare resources reimbursed by the NHS and provide information on: (i) demographics (age, sex), and vital status (accounting for all-cause mortality); (ii) drugs prescriptions, such as brand name, Anatomical-Therapeutic Chemical (ATC) code, marketing authorizations code, number of packages, number of units per package, and prescription date; this database includes the community pharmaceutical flow as well as the direct pharmaceutical distribution flow, which allows also to identify medicines dispensed by the NHS hospitals for outpatients use; (iii) hospitalizations, including discharge diagnoses coded with the International Classification of Diseases, 9th Revision, Clinical Modification (ICD-9-CM), Diagnosis Related Group (DRG) and DRG-related charge; (iv) outpatient specialist service database for information on specialist visits. To grant privacy, an anonymous univocal numeric code (Patient ID) was assigned to each subject participating in the analysis. The Patient ID allows the electronic linkage between databases and ensures the anonymity of the extracted data in full compliance with EU Data Privacy Regulation 2016/679 (‘GDPR’) and Italian D.lgs. n. 196/2003, as amended by D.lgs. n. 101/2018. All the results have been provided as aggregated data, so that they cannot identify, either directly or indirectly, specific individuals. The study was conducted in line with the principles of the Declaration of Helsinki and approved by the Ethics Committees of the participating LHU.

The data underlying this article cannot be shared publicly because they derive from the original databases of the LHU participating to the project and report personal information of patients.

### Patients and study design

Patients with a hospital discharge diagnosis of CCS (ICD-9-CM codes 413-414) between 2011 and 2020 were identified and then assigned to one of two mutually exclusive ‘ranolazine’ or ‘no-ranolazine’ cohorts, consisting respectively of patients with or without at least 1-year treatment with that agent (ATC code: C01EB18). Prescription of other drugs recommended as first-line or add-on agents by guidelines (beta-blockers, ATC code C07; calcium channel blockers, ATC code C08; nitrates, ATC code C01DA; ivabradine, ATC code C01EB17; nicorandil, ATC code C01DX16) was also recorded in all patients of both cohorts.^1^

The date of the first prescription of ranolazine or, in the no-ranolazine cohort, of one of the above-mentioned drugs during the inclusion period, was considered as the index date, according to which, age was assigned. Patients’ overall health profile was assessed over the whole period available before the index date (characterization period, at least 1-year long) and followed for the whole time after the index date (follow-up period, lasting at least 1 year). Overall health profile was characterized by the Charlson Comorbidity Index (CCI), a scoring system based on age class and on the burden of 19 concomitant conditions.^14^ The comorbidities included in CCI and other conditions of interest, were assessed during the whole period before the index date using a combination of prescribed drugs and/or hospital discharge ICD-9-CM codes, as follows: diabetes mellitus (at least one prescription of ATC code A10 drugs or a discharge code 250); dyslipidemia (at least one prescription of ATC code C10 drugs); arterial hypertension (at least one prescription of ATC codes C02, C03, C07, C08, C09); stroke (discharge codes 430–438); chronic heart failure (CHF; discharge code 428); myocardial infarction (MI; discharge codes 410–412); chronic kidney disease (CKD; at least one prescription of ATC codes A12AA, A02AH, B03XA, V03AE02, V03AE03, V03AE04, A11CC03, A11CC04 drugs, or discharge code 585); AF (discharge code 427.31); chronic obstructive pulmonary disease (COPD; at least one prescription of ATC code R03 drugs); cancer (at least one prescription of ATC code L01 drugs, or an exemption code 048, or discharge codes 140–209); cardiovascular procedures, such as coronary artery bypass grafting or other cardiac surgery, coronary angioplasty, peripheral artery angioplasty or vascular surgery, pacemaker and/or ICD implantation (discharge codes 00.4X, 00.5X, 00.6X, 35.XX-39.XX).

The primary and secondary outcomes were evaluated from 6 months after the index date over the whole period of data availability (thus omitting early events, potentially indicating clinical instability). Adherence to treatment was evaluated using the Medication Possession Ratio, calculated from prescription data in the administrative databases. Also, to ensure the analysis of a population with at least a moderate adherence to guideline-based recommendations, patients with < 3 drug prescriptions were excluded. Following an intention-to-treat principle, incident primary and secondary outcome events during the follow-up were attributed as follows: new-onset AF, diagnosed by discharge code 427.31 in patients without such a diagnosis during the characterization period; all-cause death; bradyarrhythmias, diagnosed by discharge codes 426, 427.89 or procedural code 00.5X; ventricular tachyarrhythmias, diagnosed by discharge codes 427.1 or 427.4.

### Statistical analysis

Continuous variables are presented as mean ± standard deviation (SD), and categorical variables as numbers and percentages. To check unbalance for the baseline characteristics between the study cohorts, the standardized mean difference (SMD) was computed. A SMD <0.2 was considered for the definition of variables as ‘comparable’.^15^ A propensity score matching (PSM) procedure, with a ranolazine:no-ranolazine 1:4 matching ratio that followed the initial patients’ distribution, was used to minimize the differences in baseline characteristics.

A multivariable Cox regression analysis was used to compare the risk of events between the PSM-balanced treatment cohorts. The related hazard ratios (HRs), with 95% confidence intervals (95% CI), were calculated adjusting for the following baseline variables: age, sex, CCI, diabetes, dyslipidemia, arterial hypertension, stroke, CHF, MI, CKD, AF, COPD, cancer, cardiovascular procedures, and treatment with beta-blockers, calcium channel blockers, nitrates, ivabradine, nicorandil and antithrombotic agents. Furthermore, to exclude the influence of potential confounders not included in multivariable models, we analyzed three different outcomes, which, from a pharmacologic point of view, should not have been influenced by ranolazine. We selected colorectal cancer, pulmonary embolism and urinary tract infections, representing, respectively, a neoplastic, a cardiovascular and an infective condition. In all these cases, statistical significance was identified by a *P* value <0.05. All analyses were performed using STATA SE version 17.0.

## Results

**Figure 1:**
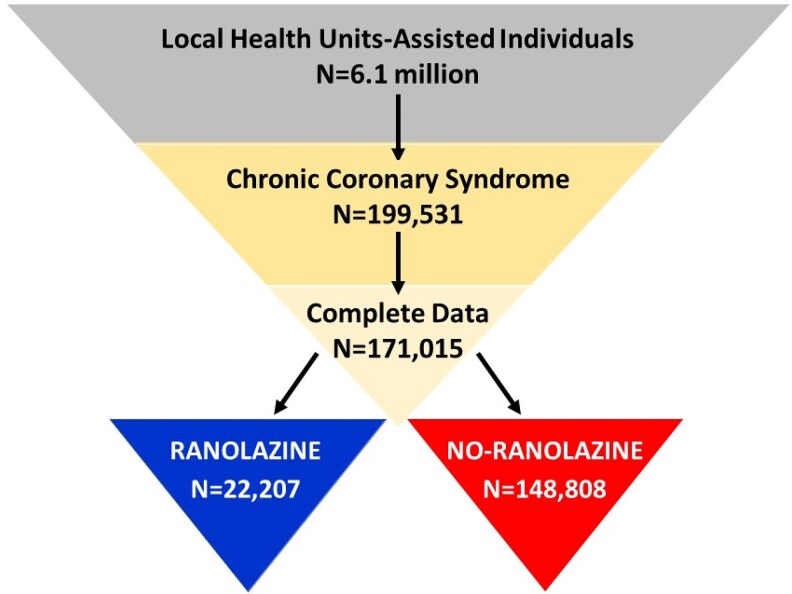
Flow-chart representing the inclusion process of patients into the study, starting from the original population enclosed in the administrative databases of the Italian local health units participating to the project.

The databases contained 199 531 patients with CCS as diagnosed using the above-cited ICD-9-CM codes. Of these, 171 015 with complete data available for at least one year before and after the index date were selected for analysis, which included 22 207 in the ranolazine and 148 808 in the no-ranolazine cohort (*Figure 1*), with an average follow-up respectively of 4.4 ± 2.6 and 5.0 ± 3.2 years (*P* < 0.001).

The demographic and clinical characteristics of the whole population and of the two treatment cohorts are reported in *Table 1*. The average age well beyond 70 years—with 28.4% aged ≥80 years—and the predominant male sex are consistent with the current epidemiological scenario. Hypertension was the most prevalent cardiovascular risk factor in both treatment cohorts, followed by dyslipidemia—which was significantly more prevalent in the ranolazine cohort—and diabetes. While the overall burden of comorbidities as expressed by the CCI, and the prevalence of previous stroke, CKD, COPD, and cancer, were similar in the two treatment cohorts, ranolazine-treated patients more frequently had had a MI and a cardiovascular procedure (*Table 1*). Prescription of pharmacological agents recommended by guidelines was similar in the two cohorts, with more than 90% of patients receiving a first-line treatment (beta- or calcium-channel blockers). After PSM, there were 6384 patients in the ranolazine and 25 536 in the no-ranolazine cohort, which were well balanced as indicated by non-significant (<0.2) SMD for all variables (*Table 2*).

**Table 1:** Demographic and clinical characteristics of the whole study population and of the ranolazine and no-ranolazine cohorts

	Whole Population *N* = 171 015	Ranolazine *N* = 22 207	No-Ranolazine *N* = 148 808	SMD
Age, years (mean ± SD)	72.0 ± 11.5	71.5 ± 10.4	72.1 ± 11.6	0.058
Sex, men (*n*, %)	112 813 (66.0)	15 007 (67.6)	97 806 (65.7)	0.033
Charlson Comorbidity Index (mean ± SD)	1.3 ± 1.4	1.2 ± 1.2	1.3 ± 1.4	0.044
Hypertension (*n*, %)	162 003 (94.7)	21 585 (97.2)	140 418 (94.4)	0.141
Dyslipidemia (*n*, %)	121 208 (70.9)	19 884 (89.5)	101 324 (68.1)	0.544
Diabetes (*n*, %)	52 290 (30.6)	8583 (38.6)	43 707 (29.4)	0.197
Atrial Fibrillation (*n*, %)	25 046 (14.6)	2519 (11.3)	22 527 (15.1)	0.112
Heart Failure (*n*, %)	30 847 (18.0)	3929 (17.7)	26 918 (18.1)	0.010
Previous Myocardial Infarction (n, %)	38 549 (22.5)	8083 (36.4)	30 466 (20.5)	0.359
Previous Stroke (*n*, %)	30 660 (17.9)	3747 (16.9)	26 913 (18.1)	0.032
CKD (*n*, %)	19 715 (11.5)	2606 (11.7)	17 109 (11.5)	0.007
COPD (*n*, %)	47 336 (27.7)	6870 (30.9)	40 466 (27.2)	0.083
Cancer (*n*, %)	14 438 (8.4)	1695 (7.6)	12 743 (8.6)	0.034
Cardiovascular Procedures (*n*, %)	77 809 (45.5)	12 450 (56.1)	65 359 (43.9)	0.245
Antithrombotics (*n*, %)	152 076 (88.9)	19 880 (89.5)	132 196 (88.8)	0.022
Beta-blockers (*n*, %)	115 365 (67.5)	15 546 (70.0)	99 819 (67.1)	0.063
Calcium Antagonists (*n*, %)	44 403 (26.0)	5250 (23.6)	39 153 (26.3)	0.062
Nitrates (*n*, %)	39 752 (23.2)	6653 (30.0)	33 099 (22.2)	0.176
Ivabradine (*n*, %)	7693 (4.5)	1713 (7.7)	5980 (4.0)	0.158

Age is at the inclusion in the study, while clinical variables refer at the pre-index, characterization, period. Antithrombotics include any anticoagulant or antiplatelet agent; Cardiovascular procedures, any of coronary artery bypass grafting or other cardiac surgery, coronary angioplasty, peripheral artery angioplasty or vascular surgery, pacemaker and or ICD implantation.

CKD, chronic kidney disease; COPD, chronic obstructive pulmonary disease; SD, standard deviation; SMD, standardized mean difference (values ≥0.2 indicate a significant difference).

**Table 2:** Demographic and clinical characteristics, and frequency of comorbidities in ranolazine and no-ranolazine cohorts after propensity score matching balancing

	Ranolazine *N* = 6384	No-Ranolazine *N* = 25 536	SMD
Age, years (mean ± SD)	70.7 ± 10.8	70.3 ± 11.8	0.030
Sex, men (*n*, %)	4419 (69.2)	17 772 (69.6)	0.008
Charlson Comorbidity Index (mean ± SD)	0.7 ± 1.0	0.7 ± 1.0	0.004
Hypertension (*n*, %)	6190 (97.0)	24 700 (96.7)	0.013
Dyslipidemia (*n*, %)	5745 (90.0)	22 629 (88.6)	0.044
Diabetes (*n*, %)	185 (2.9)	924 (3.6)	0.041
Atrial Fibrillation (*n*, %)	652 (10.2)	2511 (9.8)	0.013
Heart Failure (*n*, %)	812 (12.7)	3296 (12.9)	0.006
Previous Myocardial Infarction (*n*, %)	2251 (35.3)	8467 (33.2)	0.044
Previous Stroke (*n*, %)	851 (13.3)	3350 (13.1)	0.006
CKD (*n*, %)	472 (7.4)	1901 (7.4)	0.002
COPD (*n*, %)	1775 (27.8)	6825 (26.7)	0.024
Cancer (*n*, %)	432 (6.8)	1661 (6.5)	0.011
Cardiovascular procedures (*n*, %)	3524 (55.2)	13 701 (53.7)	0.031
Antithrombotics (*n*, %)	5802 (90.9)	22 571 (88.4)	0.082
Beta-blockers (*n*, %)	4453 (69.8)	18 041 (70.7)	0.020
Calcium Antagonists (*n*, %)	1373 (21.5)	5624 (22.0)	0.013
Nitrates (*n*, %)	1754 (27.5)	6511 (25.5)	0.045
Ivabradine (*n*, %)	383 (6.0)	1382 (5.4)	0.025

Antithrombotics include any anticoagulant or antiplatelet agent; Cardiovascular Procedures, any of coronary artery bypass grafting or other cardiac surgery, coronary angioplasty, peripheral artery angioplasty or vascular surgery, pacemaker and or ICD implantation.

CKD, chronic kidney disease; COPD, chronic obstructive pulmonary disease; SD, standard deviation; SMD, standardized mean difference (values ≥0.2 indicate a significant difference).

**Figure 2:**
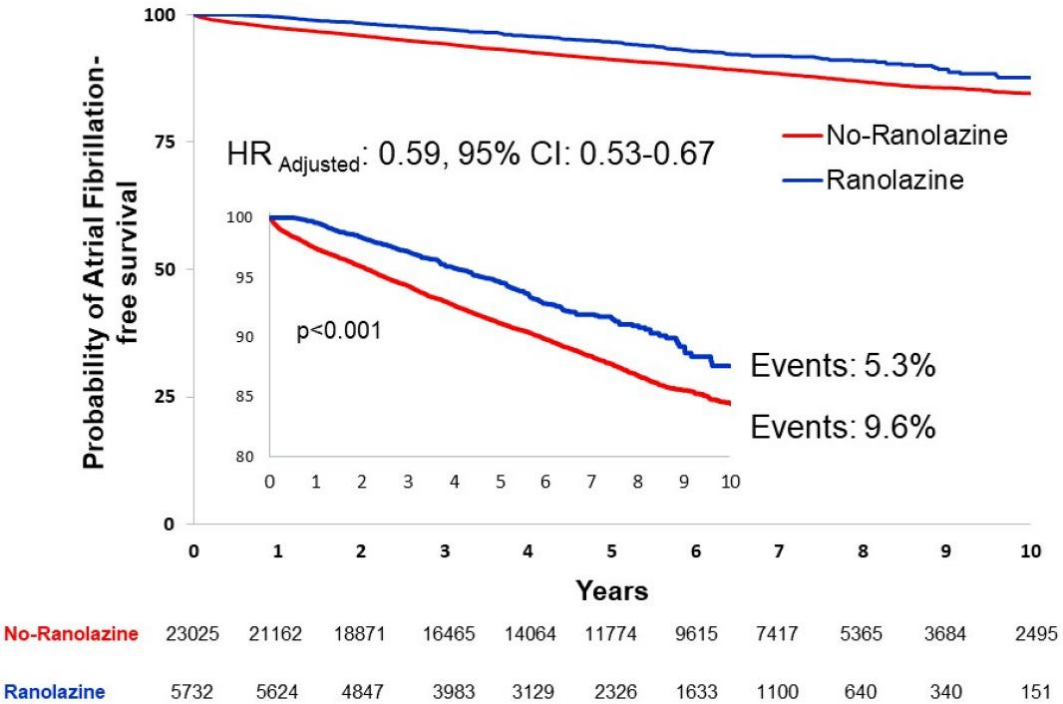
Atrial fibrillation-free survival in the ranolazine and the no-ranolazine cohorts at the Kaplan-Meier analysis. The Hazard Ratio and the related 95% confidence intervals derive from the Cox regression multivariable model.

After excluding patients who had experienced AF in the 12 months prior to enrollment, the incidence of the arrhythmia during the follow-up was significantly lower in the ranolazine than in the no-ranolazine cohort (5.3% vs. 9.6%, *P* < 0.001) (*Figure 2*). At multivariable Cox regression analysis, ranolazine was associated with a 41% reduction of the incident risk of new-onset AF (HR 0.59, 95% CI: 0.53–0.67, *P* < 0.001) (*Table 3*). Moreover, older age, male sex, heart failure, previous MI, CKD, COPD, and prescription of beta-blockers, calcium antagonists and nitrates were all predictors of an increased risk, while cardiovascular procedures and antithrombotics were associated with a decreased risk of incident AF (*Table 3*). Ranolazine treatment was also associated with a significantly lower all-cause mortality (31.7% vs. 20.0%, *P* < 0.001) and a 26% multivariable risk reduction of death (HR 0.74, 95% CI: 0.70–0.79, *P* < 0.001) (*Table 4*).

**Table 3:** Cox regression model for factors associated with risk of new-onset atrial fibrillation^a^

	HR	95% CI	*P* value
Ranolazine (vs. no-ranolazine)	0.59	0.53–0.67	<0.001
Age (for 1-year increase)	1.07	1.07–1.07	<0.001
Sex (Male vs. Female)	1.23	1.13–1.35	<0.001
Heart Failure (yes vs. no)	1.66	1.49–1.85	<0.001
Previous Myocardial Infarction (yes vs. no)	1.11	1.02–1.21	0.019
CKD (yes vs. no)	1.31	1.15–1.49	<0.001
COPD (yes vs. no)	1.18	1.08–1.28	<0.001
Cardiovascular Procedures (yes vs. no)	0.88	0.81–0.95	0.002
Antithrombotics (yes vs. no)	0.84	0.77–0.91	<0.001
Beta-blockers (yes vs. no)	1.13	1.08–1.23	0.007
Calcium Antagonists (yes vs. no)	1.11	1.01–1.21	0.030
Nitrates (yes vs. no)	1.11	1.02–1.21	0.013

^a^Patients with previous episodes of atrial fibrillation were excluded from analysis.

Antithrombotics include any anticoagulant or antiplatelet agent.

CI, confidence interval; CKD, chronic kidney disease; COPD, chronic obstructive pulmonary disease; HR, hazard ratio. Variables backward eliminated from model (*P* out ≥0.050): Charlson Comorbidity Index; hypertension; dyslipidemia; diabetes; previous stroke; neoplasms; ivabradine.

**Table 4:** Cox regression model for factors associated with risk of all-cause death

	HR	95% CI	*P*
Ranolazine (vs. no-ranolazine)	0.74	0.70–0.79	<0.001
Age (for 1-year increase)	1.09	1.08–1.09	<0.001
Sex (Male vs. Female)	1.32	1.26–1.38	<0.001
Charlson Comorbidity Index (for one-point increase)	1.22	1.19–1.25	<0.001
Dyslipidemia (yes vs. no)	0.83	0.79–0.88	<0.001
Diabetes (yes vs. no)	1.16	1.06–1.28	0.002
Atrial Fibrillation (yes vs. no)	1.20	1.13–1.27	<0.001
Heart Failure (yes vs. no)	1.60	1.52–1.69	<0.001
Previous Myocardial Infarction (yes vs. no)	1.10	1.05–1.15	<0.001
Previous Stroke (yes vs. no)	1.16	1.10–1.23	<0.001
CKD (yes vs. no)	1.32	1.24–1.40	<0.001
COPD (yes vs. no)	1.10	1.05–1.16	<0.001
Neoplasms (yes vs. no)	1.38	1.28–1.49	<0.001
Cardiovascular Procedures (yes vs. no)	0.84	0.81–0.88	<0.001
Antithrombotics (yes vs. no)	0.89	0.85–0.93	<0.001
Calcium Antagonists (yes vs. no)	0.89	0.85–0.93	<0.001
Nitrates (yes vs. no)	1.21	1.16–1.26	<0.001
Ivabradine (yes vs. no)	1.33	1.22–1.44	<0.001

Antithrombotics include any anticoagulant or antiplatelet agent.

CI, confidence interval; CKD, chronic kidney disease; COPD, chronic obstructive pulmonary disease; HR, hazard ratio.

Variables backward eliminated from model (*P* out ≥0.050): hypertension; beta-blockers.

Further, Cox regression models showed that ranolazine was also associated with a significantly lower risk of bradyarrhythmias and ventricular tachycardia/VF. In fact, bradyarrhythmias occurred respectively in 3.0% and 4.3% of the ranolazine and the no-ranolazine cohorts (*P* < 0.001), with a 24% risk reduction (HR 0.76, 95% CI: 0.65–0.89, *P* = 0.001) after adjusting for age, sex, CCI, heart failure and cardiovascular procedures (see Supplementary material online, *Table S1*). Similarly, ventricular tachycardia/VF was recorded respectively in 1.4% and 1.8% of the ranolazine and the no-ranolazine cohorts (*P* = 0.015), which corresponds to a 20% risk reduction (HR 0.80, 95% CI: 0.63–1.00, *P* = 0.049) after adjusting for sex, AF, heart failure, previous stroke, myocardial infarction, ivabradine and nitrates prescription (see Supplementary material online, *Table S2*).

Last, in the multivariable models exploring outcomes unrelated to drug action, no association was found between ranolazine and colorectal cancer (HR 0.80, 95% CI 0.57–1.12, *P* = 0.192; Supplementary material online, *Table S3*), pulmonary embolism (HR 1.15, 95% CI 0.78–1.68, *P* = 0.479; Supplementary material online, *Table S4*) and urinary tract infections (HR 0.88, 95% CI 0.71–1.10, *P* = 0.263; Supplementary material online, *Table S5*).

## Discussion

This is the first study designed to explore in a large sample of unselected, community-living individuals, the efficacy and safety of ranolazine for AF prevention in patients with CCS. Of the 171 015 patients included, 45.5% were aged ≥75 years, and more than 2/3 were men, findings that are consistent with the epidemiology of CCS in Western countries.^1,16^

In our study population, those treated with ranolazine showed a greater prevalence of cardiovascular risk factors and related conditions—in particular dyslipidemia, diabetes, and previous MI—and more frequently underwent cardiovascular procedures, such as myocardial revascularization. We can hypothesize that this observation reflects a clinicians’ attitude to prescribe ranolazine—which is known to exert favorable effects on glucose metabolism and endothelial function^17-19^—to patients perceived at greater cardiovascular risk. Such an approach may be regarded as substantially consistent with an individualized therapy based on first-line (beta- and calcium channel blockers) and add-on (ranolazine and long-acting nitrates) agents, as recommended by guidelines on the basis of symptoms control and risk factors burden.^1^

The results of the present study are consistent with a ranolazine-associated reduction in the incidence of AF, as well as of brady-arrhythmias and ventricular tachycardia. Evidence of the anti-arrhythmic properties of ranolazine derived from the MERLIN-TIMI 36 trial, which enrolled patients with non-ST segment elevation myocardial infarction (NSTEMI) undergoing continuous ECG monitoring during the first seven days after the acute event. Compared with placebo, ranolazine was associated with a significant reduction of ventricular tachycardia lasting ≥8 beats.^2^ In a subsequent post-hoc analysis of the same study, the 1-year incidence of AF, interpreted as an adverse event, was significantly reduced in ranolazine-treated patients.^20^ Moreover, when compared with amiodarone only therapy, the combination of ranolazine and amiodarone accelerated the pharmacologic cardioversion of AF.^10^ Similarly, in the HARMONY trial, the combination of dronedarone and ranolazine resulted effective in reducing the AF burden in subjects implanted with a pacemaker.^11^ Finally, a meta-analysis of five different trials showed a 53% lower risk of AF in ranolazine-treated than in placebo-treated patients.^21^ However, it is important to highlight that, of 3629 ranolazine-treated patients included in that meta-analysis, 3162 were enrollees of the MERLIN-TIMI 36 trial, while only 462 had been drawn from the other four studies.^21^ This supports the need for enriching the evidence on the anti-arrhythmic properties of ranolazine in wider populations, such as in our real-world study. Based on these experiences, ranolazine was classified as a class Id (voltage-gated Na^+^ channel blockers-late current) agent in a ‘modernized classification’ of cardiac anti-arrhythmic drugs.^22,23^ From a mechanistic point of view, studies in animal models supports the capability of ranolazine to convert paroxysmal AF to sinus rhythm,^24^ likely due to preferential blockade of the faster activation rates typically of atrial arrhythmias.^25,26^

Our data confirm the protective effect against VA observed in the MERLIN-TIMI 36 study,^2^ without an increase in brady-arrhythmias, a finding confirming a good drug safety profile. Though it can prolong the Q-T interval, particularly when combined with amiodarone, ranolazine may be appropriate as add-on therapy to shorten the Q-T interval in LQTS3 patients with a Q-T > 500 ms, with this indication formally approved in the USA.^23^

Finally, we found a remarkable, 26% reduction of all-cause mortality in ranolazine-treated patients. In a subgroup analysis of the MERLIN-TIMI 36 trial, patients treated with PCI had a 1-year reduction of recurrent ischaemia and cardiovascular death when treated with ranolazine.^27^ However, no studies assessed either the ventricular anti-arrhythmic properties of the drug or its impact on cardiovascular mortality.

Despite its pharmacological effects, a favorable risk/benefit ratio and an almost worldwide distribution, still nowadays ranolazine prescription rates show great variability by country. Our results, if confirmed, could allow a more homogeneous and proper use of the drug. Moreover, the efficacy shown in cardiac rhythm stabilization could in part explain the improved survival we found during the follow-up, because of the association of AF with all-cause and cardiovascular mortality in patients with CCS.^28^

The main strength of the present study is the large sample size of an unselected, real-world population, allowing the inclusion of individuals generally underrepresented in randomized clinical trials, such as older, multimorbid patients, leading to an improved generalizability of our findings. However, present results, obtained through an observational design, are only hypothesis generating.

Moreover, some study limitations should be acknowledged. First, our results are based on the analysis of administrative databases of the Italian NHS, which do not include accurate information on adherence to therapeutic regimen and on risk factors potentially associated with all-cause and cardiovascular mortality and incident arrhythmias, such as smoking habits, blood pressure control, and obesity. The presence itself of comorbidities relies only on administrative data possibly leading to event underestimation or misclassification. This was particularly true when coding for cardiovascular death, which was consequently left out from the analysis. However, in Italy the access to health care is universal, and the related administrative information is present and available for the whole population. All patient’s records can be easily and reliably linked to each other through a unique individual identifier. Indeed, the validity and the accuracy of the data stored in the administrative database have been previously shown.^29^ Second, the burden of comorbidities was estimated from data antecedent to the index event determining the inclusion into the study, based on discharge diagnoses and on proxies, such as prescription of disease-specific medications and/or related hospitalizations. Also, given that data originated from administrative sources, it was impossible to graduate treatment effects by the severity of pre-existing comorbid conditions. Third, there was a small, though significant, longer duration of follow-up in the no-ranolazine cohort, a condition possibly accentuating the benefits of ranolazine therapy; this, however, should not be the case, provided the entity of the length difference (about 12%) and, particularly, the early and progressive separation of the event-free survival as depicted by the Kaplan-Meier curves outlined in *Figure 2*. The lower duration of follow-up in the ranolazine cohort might be in part attributable to the fact that, despite drug approval in Europe in 2008, the first ESC guidelines giving a clear recommendation for ranolazine therapy in patients with stable coronary artery disease (Class of recommendation: IIa, Level of evidence: B) were published in 2013.^30^ Fourth, no sensitivity analysis was performed by treatment duration. Fifth, the PSM techniques were used to balance characteristics of treatment cohorts, a method that, though well validated, is known to be at risk of not accounting for all potential confounders. Indeed, we found a lower prevalence of diabetes after the procedure. This result did not prevent, however, to show the significant association between diabetes itself and all-cause mortality in the Cox multivariable regression model (*Table 4*). Last, because of the nature of the present study, it is impossible to attribute the benefits we observed to the electrophysiologic, the metabolic and/or the anti-ischemic properties of ranolazine. If the effects which were found will be confirmed, experimental and clinical studies should have to clarify the involved mechanisms.

## Conclusions

Present analysis shows that ranolazine therapy is possibly associated with a protective long-term effect against occurrence of AF in patients with chronic coronary syndromes. The burden of bradyarrhythmias and of major VA seems not to be increased during drug treatment, while all-cause mortality appears to be reduced. These results, obtained in a real-world, epidemiological setting, press for new, specifically addressed, clinical trials, aimed at confirming the anti-arrhythmic properties of ranolazine in coronary and non-coronary patients, possibly widening therapeutic options with a drug safe and characterized by multiple, complementary, pharmacologic actions.

## Supplementary Material

pvaf074_Supplementary_Data

## Data Availability

The data underlying this article cannot be shared publicly because they derive from the original databases of the local health units participating to the project and report personal information of patients.

## References

[1] Vrints C, Andreotti F, Koskinas KC, Rossello X, Adamo M, Ainslie J, Banning AP, Budaj A, Buechel RR, Chiariello GA, Chieffo A, Christodorescu RM, Deaton C, Doenst T, Jones HW, Kunadian V, Mehilli J, Milojevic M, Piek JJ, Pugliese F, Rubboli A, Semb AG, Senior R, Ten Berg JM, Van Belle E, Van Craenenbroeck EM, Vidal-Perez R, Winther S; ESC Scientific Document Group. 2024 ESC guidelines for the management of chronic coronary syndromes. Eur Heart J 2024;45:3415–3537. 39210710 10.1093/eurheartj/ehae177

[2] Scirica BM, Morrow DA, Hod H, Murphy SA, Belardinelli L, Hedgepeth CM, Molhoek P, Verheugt FW, Gersh BJ, McCabe CH, Braunwald E. Effect of ranolazine, an antianginal agent with novel electrophysiological properties, on the incidence of arrhythmias in patients with non ST-segment elevation acute coronary syndrome: results from the metabolic efficiency with ranolazine for less ischemia in non ST-elevation acute coronary syndrome thrombolysis in myocardial infarction 36 (MERLIN-TIMI 36) randomized controlled trial. Circulation 2007;116:1647–1652. 17804441 10.1161/CIRCULATIONAHA.107.724880

[3] Kourampi I, Katsioupa M, Oikonomou E, Tsigkou V, Marinos G, Goliopoulou A, Katsarou O, Kalogeras K, Theofilis P, Tsatsaragkou A, Siasos G, Tousoulis D, Vavuranakis M. The role of ranolazine in heart failure-current concepts. Am J Cardiol 2023;209:92–103. 37844876 10.1016/j.amjcard.2023.09.066

[4] Fraser H, Belardinelli L, Wang L, Light PE, McVeigh JJ, Clanachan AS. Ranolazine decreases diastolic calcium accumulation caused by ATX-II or ischemia in rat hearts. J Mol Cell Cardiol 2006;41:1031–1038. 17027025 10.1016/j.yjmcc.2006.08.012

[5] Nash DT, Nash SD. Ranolazine for chronic stable angina. Lancet 2008;372:1335–1341. 18929905 10.1016/S0140-6736(08)61554-8

[6] Rouhana S, Virsolvy A, Fares N, Richard S, Thireau J. Ranolazine: an old drug with emerging potential; lessons from Pre-clinical and clinical investigations for possible repositioning. Pharmaceuticals (Basel) 2021;15:32–36. 35056088 10.3390/ph15010031 PMC8777683

[7] Ratte A, Wiedmann F, Kraft M, Katus HA, Schmidt C. Antiarrhythmic properties of ranolazine: inhibition of atrial fibrillation associated TASK-1 potassium channels. Front Pharmacol 2019;10:1367. 32038227 10.3389/fphar.2019.01367 PMC6988797

[8] Verrier RL, Kumar K, Nieminen T, Belardinelli L. Mechanisms of ranolazine's dual protection against atrial and ventricular fibrillation. Europace 2013;15:317–324. 23220484 10.1093/europace/eus380 PMC3578672

[9] De Ferrari GM, Maier LS, Mont L, Schwartz PJ, Simonis G, Leschke M, Gronda E, Boriani G, Darius H, Guillamón Torán L, Savelieva I, Dusi V, Marchionni N, Quintana Rendón M, Schumacher K, Tonini G, Melani L, Giannelli S, Alberto Maggi C, Camm AJ; RAFFAELLO Investigators (see Online Supplementary Appendix for List of Participating Centers and Investigators). Ranolazine in the treatment of atrial fibrillation: results of the dose-ranging RAFFAELLO (ranolazine in atrial fibrillation following an ELectricaL CardiOversion) study. Heart rhythm 2015;12:872–878. 25602175 10.1016/j.hrthm.2015.01.021

[10] Tsanaxidis N, Aidonidis I, Hatziefthimiou A, Daskalopoulou SS, Giamouzis G, Triposkiadis F, Skoularigis I. Ranolazine added to amiodarone facilitates earlier conversion of atrial fibrillation compared to amiodarone-only therapy. Pacing Clin Electrophysiol 2017;40:372–378. 28182279 10.1111/pace.13048

[11] Reiffel JA, Camm AJ, Belardinelli L, Zeng D, Karwatowska-Prokopczuk E, Olmsted A, Zareba W, Rosero S, Kowey P; HARMONY Investigators. The HARMONY trial: combined ranolazine and dronedarone in the management of paroxysmal atrial fibrillation: mechanistic and therapeutic synergism. Circ Arrhythm Electrophysiol 2015;8:1048–1056. 26226999 10.1161/CIRCEP.115.002856

[12] White CM, Nguyen E. Novel use of ranolazine as an antiarrhythmic agent in atrial fibrillation. Ann Pharmacother 2017;51:245–252. 27758968 10.1177/1060028016673073

[13] Bo M, Fumagalli S, Degli Esposti L, Perrone V, Dovizio M, Poli D, Marcucci R, Verdecchia P, Reboldi G, Lip GYH, Ungar A, Boccanelli A, Fumagalli C, Marchionni N; Italian Society of Geriatric Cardiology (SICGe). Anticoagulation in atrial fibrillation. A large real-world update. Eur J Intern Med 2024;121:88–94. 37879969 10.1016/j.ejim.2023.10.010

[14] Charlson ME, Pompei P, Ales KL, MacKenzie CR. A new method of classifying prognostic comorbidity in longitudinal studies: development and validation. J Chronic Dis 1987;40:373–383. 3558716 10.1016/0021-9681(87)90171-8

[15] Andrade C . Mean difference, standardized mean difference (SMD), and their use in meta-analysis: as simple as it gets. J Clin Psychiatry 2020;81:20f13681. 10.4088/JCP.20f13681 32965803

[16] Martin SS, Aday AW, Almarzooq ZI, Anderson CAM, Arora P, Avery CL, Baker-Smith CM, Barone Gibbs B, Beaton AZ, Boehme AK, Commodore-Mensah Y, Currie ME, Elkind MSV, Evenson KR, Generoso G, Heard DG, Hiremath S, Johansen MC, Kalani R, Kazi DS, Ko D, Liu J, Magnani JW, Michos ED, Mussolino ME, Navaneethan SD, Parikh NI, Perman SM, Poudel R, Rezk-Hanna M, Roth GA, Shah NS, St-Onge MP, Thacker EL, Tsao CW, Urbut SM, Van Spall HGC, Voeks JH, Wang NY, Wong ND, Wong SS, Yaffe K, Palaniappan LP; American Heart Association Council on Epidemiology and Prevention Statistics Committee and Stroke Statistics Subcommittee. 2024 heart disease and stroke statistics: a report of US and global data from the American Heart Association. Circulation 2024;149:e347–e913. 38264914 10.1161/CIR.0000000000001209 PMC12146881

[17] Timmis AD, Chaitman BR, Crager M. Effects of ranolazine on exercise tolerance and HbA1c in patients with chronic angina and diabetes. Eur Heart J 2006;27:42–48. 16176940 10.1093/eurheartj/ehi495

[18] Morrow DA, Scirica BM, Chaitman BR, McGuire DK, Murphy SA, Karwatowska-Prokopczuk E, McCabe CH, Braunwald E; MERLIN-TIMI 36 Investigators. Evaluation of the glycometabolic effects of ranolazine in patients with and without diabetes mellitus in the MERLIN-TIMI 36 randomized controlled trial. Circulation 2009;119:2032–2039. 19349325 10.1161/CIRCULATIONAHA.107.763912

[19] Nusca A, Bernardini F, Mangiacapra F, Maddaloni E, Melfi R, Ricottini E, Piccirillo F, Manfrini S, Ussia GP, Grigioni F. Ranolazine improves glycemic variability and endothelial function in patients with diabetes and chronic coronary syndromes: results from an experimental study. J Diabetes Res 2021;2021:4952447. 35005029 10.1155/2021/4952447 PMC8741377

[20] Scirica BM, Belardinelli L, Chaitman BR, Waks JW, Volo S, Karwatowska-Prokopczuk E, Murphy SA, Cheng ML, Braunwald E, Morrow DA. Effect of ranolazine on atrial fibrillation in patients with non-ST elevation acute coronary syndromes: observations from the MERLIN-TIMI 36 trial. Europace 2015;17:32–37. 25210025 10.1093/europace/euu217

[21] Guerra F, Romandini A, Barbarossa A, Belardinelli L, Capucci A. Ranolazine for rhythm control in atrial fibrillation: a systematic review and meta-analysis. Int J Cardiol 2017;227:284–291. 27839812 10.1016/j.ijcard.2016.11.103

[22] Lei M, Wu L, Terrar DA, Huang CL. Modernized classification of cardiac antiarrhythmic drugs. Circulation 2018;138:1879–1896. 30354657 10.1161/CIRCULATIONAHA.118.035455

[23] Merino JL, Tamargo J, Blomström-Lundqvist C, Boriani G, Crijns HJGM, Dobrev D, Goette A, Hohnloser SH, Naccarelli GV, Reiffel JA, Tfelt-Hansen J, Martínez-Cossiani M, Camm AJ, Almendral Garrote JM, Średniawa B, Kułakowski P, Savelieva I, Potpara T, Gorenek B, Zamorano JL. Practical compendium of antiarrhythmic drugs: a clinical consensus statement of the European heart rhythm association of the ESC. Europace 2025;27:euaf076. 40159403 10.1093/europace/euaf076 PMC12367031

[24] Ramirez RJ, Takemoto Y, Martins RP, Filgueiras-Rama D, Ennis SR, Mironov S, Bhushal S, Deo M, Rajamani S, Berenfeld O, Belardinelli L, Jalife J, Pandit SV. Mechanisms by which ranolazine terminates paroxysmal but not persistent atrial fibrillation. Circ Arrhythm Electrophysiol 2019;12:e005557. 31594392 10.1161/CIRCEP.117.005557 PMC6788778

[25] Burashnikov A, Di Diego JM, Zygmunt AC, Belardinelli L, Antzelevitch C. Atrium-selective sodium channel block as a strategy for suppression of atrial fibrillation: differences in sodium channel inactivation between atria and ventricles and the role of ranolazine. Circulation 2007;116:1449–1457. 17785620 10.1161/CIRCULATIONAHA.107.704890 PMC2566303

[26] Zygmunt AC, Nesterenko VV, Rajamani S, Hu D, Barajas-Martinez H, Belardinelli L, Antzelevitch C. Mechanisms of atrial-selective block of Na(+) channels by ranolazine: I. Experimental analysis of the use-dependent block. Am J Physiol Heart Circ Physiol 2011;301:H1606–H1614. 21821778 10.1152/ajpheart.00242.2011 PMC3197375

[27] Gutierrez JA, Karwatowska-Prokopczuk E, Murphy SA, Belardinelli L, Farzaneh-Far R, Walker G, Morrow DA, Scirica BM. Effects of ranolazine in patients with chronic angina in patients with and without percutaneous coronary intervention for acute coronary syndrome: observations from the MERLIN-TIMI 36 trial. Clin Cardiol 2015;38:469–475. 26059896 10.1002/clc.22425 PMC6711055

[28] Kerneis M, Cosentino F, Ferrari R, Georges JL, Kosmachova E, Laroche C, Maggioni AP, Rittger H, Steg PG, Maczynska J, Tavazzi L, Valgimigli M, Gale CP, Komajda M; CICD investigators group. Impact of chronic coronary syndromes on cardiovascular hospitalization and mortality: the ESC-EORP CICD-LT registry. Eur J Prev Cardiol 2022;29:1945–1954. 35653582 10.1093/eurjpc/zwac089

[29] Degli Esposti L, Perrone V, Veronesi C, Buda S, Rossini R. All-cause mortality, cardiovascular events, and health care costs after 12 months of dual platelet aggregation inhibition after acute myocardial infarction in real-world patients: findings from the platelet-aggregation inhibition: persistence with treatment and cardiovascular events in real world (PIPER) study. Vasc Health Risk Manag 2018;14:383–392. 30538488 10.2147/VHRM.S162004 PMC6251357

[30] Task Force Members; Montalescot G, Sechtem U, Achenbach S, Andreotti F, Arden C, Budaj A, Bugiardini R, Crea F, Cuisset T, Di Mario C, Ferreira JR, Gersh BJ, Gitt AK, Hulot JS, Marx N, Opie LH, Pfisterer M, Prescott E, Ruschitzka F, Sabaté M, Senior R, Taggart DP, van der Wall EE, Vrints CJ; ESC Committee for Practice Guidelines; Zamorano JL, Achenbach S, Baumgartner H, Bax JJ, Bueno H, Dean V, Deaton C, Erol C, Fagard R, Ferrari R, Hasdai D, Hoes AW, Kirchhof P, Knuuti J, Kolh P, Lancellotti P, Linhart A, Nihoyannopoulos P, Piepoli MF, Ponikowski P, Sirnes PA, Tamargo JL, Tendera M, Torbicki A, Wijns W, Windecker S; Document Reviewers; Knuuti J, Valgimigli M, Bueno H, Claeys MJ, Donner-Banzhoff N, Erol C, Frank H, Funck-Brentano C, Gaemperli O, Gonzalez-Juanatey JR, Hamilos M, Hasdai D, Husted S, James SK, Kervinen K, Kolh P, Kristensen SD, Lancellotti P, Maggioni AP, Piepoli MF, Pries AR, Romeo F, Rydén L, Simoons ML, Sirnes PA, Steg PG, Timmis A, Wijns W, Windecker S, Yildirir A, Zamorano JL. 2013 ESC guidelines on the management of stable coronary artery disease: the task force on the management of stable coronary artery disease of the European Society of Cardiology. Eur Heart J 2013;34:2949–3003. 23996286 10.1093/eurheartj/eht296

