# Supplementary material for: Ranolazine in patients with chronic coronary syndromes: real-world data provide new evidence on the antiarrhythmic properties of the drug
Source: Eur Heart J Cardiovasc Pharmacother. 2025 Sep 30;11(8):674–81. doi: 10.1093/ehjcvp/pvaf074 (PMC12705168; doi:10.1093/ehjcvp/pvaf074)
Supplement: pvaf074_Supplementary_Data [file pvaf074_supplementary_data.docx]

**Supplementary Material**

**Supplementary Table 1.** Cox regression model for factors associated with the risk of bradyarrhythmia

|  | **HR** | **95% CI** | **P value** |
| --- | --- | --- | --- |
| Ranolazine (vs. no ranolazine) | 0.76 | 0.65 - 0.88 | <0.001 |
| Age (for one-year increase) | 1.04 | 1.03 - 1.04 | <0.001 |
| Sex (Male vs. Female) | 1.56 | 1.37 - 1.77 | <0.001 |
| Charlson Comorbidity Index (for one-point increase) | 1.09 | 1.01 - 1.18 | 0.035 |
| Heart failure (yes vs. no) | 2.06 | 1.79 - 2.38 | <0.001 |
| Ivabradine (yes vs. no) | 1.26 | 1.01 - 1.57 | 0.045 |

Abbreviations: CI, confidence interval; CKD, chronic kidney disease; COPD, chronic obstructive pulmonary disease; HR, hazard ratio. Variables backward eliminated from model (p out >0.050): antithrombotics, atrial fibrillation, beta-blockers, calcium antagonists, cancer, cardiovascular procedures, CKD, COPD, diabetes, dyslipidemia, hypertension, nitrates, previous myocardial infarction, previous stroke

**Supplementary Table 2.** Cox regression model for factors associated with the risk of ventricular tachycardia/ventricular fibrillation

|  | **HR** | **95% CI** | **p** |
| --- | --- | --- | --- |
| Ranolazine (vs. no ranolazine) | 0.79 | 0.63 - 0.99 | 0.042 |
| Sex (Male vs. Female) | 3.16 | 2.45 - 4.07 | <0.001 |
| Atrial fibrillation (yes vs. no) | 1.38 | 1.07 - 1.77 | 0.013 |
| Heart failure (yes vs. no) | 3.52 | 2.90 - 4.27 | <0.001 |
| Previous Myocardial Infarction (yes vs. no) | 1.39 | 1.17 - 1.66 | <0.001 |
| Nitrates (yes vs. no) | 1.29 | 1.07 - 1.55 | 0.008 |
| Ivabradine (yes vs. no) | 1.75 | 1.32 - 2.33 | <0.001 |

Abbreviations: CI, confidence interval; CKD, chronic kidney disease; COPD, chronic obstructive pulmonary disease; HR, hazard ratio. Variables backward eliminated from model (p out >0.050): age, antithrombotics, beta-blockers, calcium antagonists, cancer, cardiovascular procedures, Charlson Comorbidity Index, CKD, COPD, diabetes, dyslipidemia, hypertension, nitrates, previous stroke

**Supplementary Table 3.** Cox regression model for factors associated with the risk of colorectal cancer*

|  | **HR** | **95% CI** | **p** |
| --- | --- | --- | --- |
| Ranolazine (vs. no ranolazine)** | 0.80 | 0.57 - 1.12 | 0.192 |
| Sex (male vs. female) | 1.88 | 1.32 - 2.69 | 0.001 |
| Age | 1.05 | 1.03 - 1.07 | <0.001 |

*ICD-9 codes for diagnosis: 153, 154; **Ranolazine, with the related HR (95% CI) and P value, was reported in the table to show the absence of statistical association with the outcome variable

Abbreviations: CI, confidence interval; CKD, chronic kidney disease; COPD, chronic obstructive pulmonary disease; HR, hazard ratio. Variables backward eliminated from model (p out >0.050): antithrombotics; atrial fibrillation; cancer; cardiovascular procedures; Charlson Comorbidity Index; CKD; COPD; diabetes; dyslipidemia; heart failure; hypertension; ivabradine; previous myocardial infarction; previous stroke

**Supplementary Table 4.** Cox regression model for factors associated with the risk of pulmonary embolism*

|  | **HR** | **95% CI** | **p** |
| --- | --- | --- | --- |
| Ranolazine (vs. no ranolazine)** | 1.15 | 0.78 - 1.68 | 0.479 |
| Sex (male vs. female) | 0.63 | 0.45 - 0.90 | 0.011 |
| Age | 1.05 | 1.03 - 1.07 | <0.001 |

*ICD-9 code for diagnosis: 415.1; **Ranolazine, with the related HR (95% CI) and P value, was reported in the table to show the absence of statistical association with the outcome variable

Abbreviations: CI, confidence interval; CKD, chronic kidney disease; COPD, chronic obstructive pulmonary disease; HR, hazard ratio. Variables backward eliminated from model (p out >0.050): antithrombotics; atrial fibrillation; cancer; cardiovascular procedures; Charlson Comorbidity Index; CKD; COPD; diabetes; dyslipidemia; heart failure; hypertension; ivabradine; previous myocardial infarction; previous stroke

**Supplementary Table 5.** Cox regression model for factors associated with the risk of urinary tract infections*

|  | **HR** | **95% CI** | **p** |
| --- | --- | --- | --- |
| Ranolazine (vs. no ranolazine)** | 0.88 | 0.71 - 1.10 | 0.263 |
| Sex (male vs. female) | 0.69 | 0.57 - 0.84 | <0.001 |
| Age | 1.07 | 1.06 - 1.08 | <0.001 |
| Dyslipidemia (yes vs. no) | 0.68 | 0.53 - 0.88 | 0.003 |
| Heart failure (yes vs. no) | 1.65 | 1.29 - 2.11 | <0.001 |
| Previous stroke (yes vs. no) | 1.39 | 1.07 - 1.80 | 0.014 |
| CKD (yes vs. no) | 1.38 | 1.01 - 1.88 | 0.043 |
| Cancer (yes vs. no) | 1.68 | 1.20 - 2.36 | 0.003 |
| Previous myocardial infarction (yes vs. no) | 1.25 | 1.02 - 1.54 | 0.030 |
| Ivabradine (yes vs. no) | 1.55 | 1.10 - 2.18 | 0.012 |

*ICD-9 codes for diagnosis: 590, 599.0, 597.80; **Ranolazine, with the related HR (95% CI) and P value, was reported in the table to show the absence of statistical association with the outcome variable

Abbreviations: CI, confidence interval; CKD, chronic kidney disease; COPD, chronic obstructive pulmonary disease; HR, hazard ratio. Variables backward eliminated from model (p out >0.050): antithrombotics; atrial fibrillation; cardiovascular procedures; Charlson Comorbidity Index; COPD; diabetes; hypertension
